# Supplementary material for: Bacterial contribution to genesis of the novel germ line determinant oskar
Source: eLife. 2020 Feb 24;9:e45539. doi: 10.7554/eLife.45539 (PMC7250577; doi:10.7554/eLife.45539)
Supplement: Supplementary file 1. — (A) List of genomes and transcriptomes used for automated oskar search. (B) List of Oskar sequences used in the final alignment. (C) List of sequences used for phylogenetic analysis of the LOTUS domain. (D) List of sequences used for phylogenetic analysis of the OSK domain. [file elife-45539-supp1.pdf]

Supplementary File 1 for

<u>Bacterial contribution to genesis of the novel germ line determinant *oskar*</u>

Leo Blondel, Tamsin E. M. Jones and Cassandra G. Extavour

The Supplementary Information for this paper consists of the following elements:

Supplementary File 1

THIS PDF DOCUMENT: FILE NAME BLONDEL_JONES_EXTAVOUR_HGT_ SUPPLEMENTARY_FILE_1

1. <u>Supplementary Tables</u>
    a. Supplementary File 1A: List of genomes and transcriptomes used for automated *oskar* search.
    b. Supplementary File 1B: List of *oskar* sequences used in the final alignment.
    c. Supplementary File 1C: List of sequences used for phylogenetic analysis of the LOTUS domain.
    d. Supplementary File 1D: List of sequences used for phylogenetic analysis of the OSK domain.
    e. Supplementary File 1E: List of genomes analyzed for codon use.

Source Data 1

ZIPPED FOLDER: DOWNLOAD HERE

https://www.dropbox.com/sh/zqf6kpo0kzav7xp/AAC5WPVm9lrDrZHqZg_RlsiTa?dl=0

1. Subfolder **Alignments**: All sequences identified and analyzed in this study, in FASTA format and with corresponding Alignments
2. Subfolder **BLAST search results**: Results of BLASTP searches with full length Oskar, OSK or LOTUS domains as queries
3. Subfolder **Data**: Necessary files for running the different IPython notebooks:
    a. Subfolder ***HMM***: HMM models used for iterative searching for sequences similar to full-length Oskar, LOTUS and OSK domains
    b. Subfolder ***Taxonomy***: Conversion table for UniProt ID to taxon information. (uniprot_ID_taxa.tsv)
    c. Subfolder ***Trees***: Contains the tree files obtained from
        i. RaxML phylogenetic analyses of the OSK and LOTUS domains aligned with MUSCLE, T-Coffee or PRANK
        ii. MrBayes phylogenetic analyses of the OSK and LOTUS domains aligned with MUSCLE
        iii. SOWHAT analyses.

Scripts

All scripts used herein are hosted on GitHub at <u>https://github.com/extavourlab/Oskar_HGT</u>

Supplementary File 1A: List of genomes and transcriptomes used for automated *oskar* search. List of genomes and transcriptomes that were downloaded, annotated, and searched for *oskar* sequences (*see "Hidden Markov Model (HMM) generation and alignments of the OSK and LOTUS domains*" in Methods). The table reports the database provenance (NCBI genome or TSA, or 1KITE database) and the accession number. The TSA accession ID can be searched using the NCBI TSA browser here: https://www.ncbi.nlm.nih.gov/Traces/wgs/?view=TSA.

Type	Accession ID	Organism Name
Genome	GCA_000001765.2	*Drosophila pseudoobscura pseudoobscura*
Genome	GCA_000002325.2	*Nasonia vitripennis*
Genome	GCA_000002335.2	*Tribolium castaneum*
Genome	GCA_000004775.1	*Nasonia giraulti*
Genome	GCA_000004795.1	*Nasonia longicornis*
Genome	GCA_000005115.1	*Drosophila ananassae*
Genome	GCA_000005135.1	*Drosophila erecta*
Genome	GCA_000005195.1	*Drosophila persimilis*
Genome	GCA_000005245.1	*Drosophila virilis*
Genome	GCA_000005925.1	*Drosophila willistoni*
Genome	GCA_000005975.1	*Drosophila yakuba*
Genome	GCA_000006295.1	*Pediculus humanus corporis*
Genome	GCA_000143395.2	*Atta cephalotes*
Genome	GCA_000147175.1	*Camponotus floridanus*
Genome	GCA_000147195.1	*Harpegnathos saltator*
Genome	GCA_000149185.1	*Mayetiola destructor*
Genome	GCA_000151625.1	*Bombyx mori*
Genome	GCA_000151715.1	*Bombyx mori*
Genome	GCA_000181055.3	*Rhodnius prolixus*
Genome	GCA_000184785.1	*Apis florea*
Genome	GCA_000187875.1	*Daphnia pulex*
Genome	GCA_000187915.1	*Pogonomyrmex barbatus*
Genome	GCA_000188075.1	*Solenopsis invicta*
Genome	GCA_000209185.1	*Culex quinquefasciatus*
Genome	GCA_000211455.3	*Anopheles darlingi*
Genome	GCA_000214255.1	*Bombus terrestris*
Genome	GCA_000217595.1	*Linepithema humile*
Genome	GCA_000220665.2	*Drosophila ficusphila*
Genome	GCA_000220905.1	*Megachile rotundata*
Genome	GCA_000224215.2	*Drosophila kikkawai*
Genome	GCA_000224235.2	*Drosophila takahashii*
Genome	GCA_000233415.2	*Drosophila biarmipes*
Genome	GCA_000235995.1	*Danaus plexippus plexippus*
Genome	GCA_000236305.2	*Drosophila rhopaloa*

Genome	GCA_000239435.1	*Tetranychus urticae*
Genome	GCA_000239455.1	*Strigamia maritima*
Genome	GCA_000259055.1	*Drosophila simulans*
Genome	GCA_000262585.1	*Manduca sexta*
Genome	GCA_000262795.1	*Phlebotomus papatasi*
Genome	GCA_000265325.1	*Lutzomyia longipalpis*
Genome	GCA_000269505.2	*Drosophila miranda*
Genome	GCA_000281935.1	*Mengenilla moldrzyki*
Genome	GCA_000298335.1	*Drosophila albomicans*
Genome	GCA_000325945.1	*Plutella xylostella*
Genome	GCA_000330985.1	*Plutella xylostella*
Genome	GCA_000341935.1	*Cephus cinctus*
Genome	GCA_000344095.1	*Athalia rosae*
Genome	GCA_000347755.1	*Ceratitis capitata*
Genome	GCA_000349025.1	*Anopheles minimus*
Genome	GCA_000349045.1	*Anopheles stephensi*
Genome	GCA_000349065.1	*Anopheles quadriannulatus*
Genome	GCA_000349085.1	*Anopheles funestus*
Genome	GCA_000349105.1	*Anopheles epiroticus*
Genome	GCA_000349145.1	*Anopheles dirus*
Genome	GCA_000349165.1	*Anopheles christyi*
Genome	GCA_000349185.1	*Anopheles arabiensis*
Genome	GCA_000371365.1	*Musca domestica*
Genome	GCA_000439205.1	*Anopheles nili*
Genome	GCA_000441895.2	*Anopheles sinensis*
Genome	GCA_000469605.1	*Apis dorsata*
Genome	GCA_000472105.1	*Drosophila suzukii*
Genome	GCA_000473185.1	*Anopheles maculatus*
Genome	GCA_000473375.1	*Anopheles culicifacies*
Genome	GCA_000473445.2	*Anopheles farauti*
Genome	GCA_000473505.1	*Anopheles atroparvus*
Genome	GCA_000473525.2	*Anopheles melas*
Genome	GCA_000473845.2	*Anopheles merus*
Genome	GCA_000475195.1	*Diaphorina citri*
Genome	GCA_000500325.1	*Leptinotarsa decemlineata*
Genome	GCA_000503995.1	*Ceratosolen solmsi marchali*
Genome	GCA_000507165.1	*Ephemera danica*
Genome	GCA_000516895.1	*Locusta migratoria*
Genome	GCA_000599845.1	*Trichogramma pretiosum*
Genome	GCA_000611835.1	*Ooceraea biroi*
Genome	GCA_000648655.1	*Copidosoma floridanum*
Genome	GCA_000648675.1	*Cimex lectularius*

Genome	GCA_000648695.1	*Onthophagus taurus*
Genome	GCA_000648945.1	*Limnephilus lunatus*
Genome	GCA_000671735.1	*Glossina fuscipes fuscipes*
Genome	GCA_000688715.1	*Glossina pallidipes*
Genome	GCA_000688735.1	*Glossina austeni*
Genome	GCA_000695345.1	*Bactrocera tryoni*
Genome	GCA_000695645.1	*Pachypsylla venusta*
Genome	GCA_000696155.1	*Zootermopsis nevadensis*
Genome	GCA_000696205.1	*Oncopeltus fasciatus*
Genome	GCA_000696795.1	*Halyomorpha halys*
Genome	GCA_000696855.1	*Homalodisca vitripennis*
Genome	GCA_000699065.1	*Lucilia cuprina*
Genome	GCA_000762945.1	*Blattella germanica*
Genome	GCA_000775305.1	*Belgica antarctica*
Genome	GCA_000786065.1	*Piezodorus guildinii*
Genome	GCA_000786525.1	*Chironomus tentans*
Genome	GCA_000789215.2	*Bactrocera dorsalis*
Genome	GCA_000818775.1	*Glossina palpalis gambiensis*
Genome	GCA_000836235.1	*Papilio xuthus*
Genome	GCA_000934665.1	*Catajapyx aquilonaris*
Genome	GCA_000956155.1	*Cotesia vestalis*
Genome	GCA_000956235.1	*Wasmannia auropunctata*
Genome	GCA_000956255.1	*Anopheles punctulatus*
Genome	GCA_000956275.1	*Anopheles koliensis*
Genome	GCA_001014415.1	*Phortica variegata*
Genome	GCA_001014435.1	*Mayetiola destructor*
Genome	GCA_001014445.1	*Scaptodrosophila lebanonensis*
Genome	GCA_001014505.1	*Chironomus riparius*
Genome	GCA_001014625.1	*Bactrocera oleae*
Genome	GCA_001014835.1	*Lucilia sericata*
Genome	GCA_001014935.1	*Liriomyza trifolii*
Genome	GCA_001014945.1	*Clogmia albipunctata*
Genome	GCA_001015115.1	*Eutreta diana*
Genome	GCA_001015145.1	*Eristalis dimidiata*
Genome	GCA_001015175.1	*Megaselia abdita*
Genome	GCA_001015215.1	*Holcocephala fusca*
Genome	GCA_001015235.1	*Sphyracephala brevicornis*
Genome	GCA_001015335.1	*Stomoxys calcitrans*
Genome	GCA_001017275.1	*Calliphora vicina*
Genome	GCA_001017455.1	*Neobellieria bullata*
Genome	GCA_001017515.1	*Tephritis californica*
Genome	GCA_001017525.1	*Teleopsis dalmanni*

Genome	GCA_001017535.1	*Tipula oleracea*
Genome	GCA_001077435.1	*Glossina morsitans morsitans*
Genome	GCA_001186385.1	*Diuraphis noxia*
Genome	GCA_001188975.2	*Bactrocera oleae*
Genome	GCA_001272555.1	*Dufourea novaeangliae*
Genome	GCF_000142985.2	*Acyrthosiphon pisum*
Genome	GCF_000208615.1	*Ixodes scapularis*
Genome	GCF_000004015.3	*Aedes aegypti*
TSA	GBKU	*Trichoplusia ni*
TSA	GBRL	*Sitodiplosis mosellana*
TSA	GAAX	*Forficula auricularia*
TSA	GACV	*Philopotamus ludificatus*
TSA	GADM	*Drosophila malerkotliana malerkotliana*
TSA	GAEO	*Pissodes strobi*
TSA	GAFI	*Dendroctonus frontalis*
TSA	GAFR	*Polistes canadensis*
TSA	GAHN	*Drosophila serrata*
TSA	GAHP	*Ostrinia nubilalis*
TSA	GAHQ	*Ostrinia scapulalis*
TSA	GAIW	*Ganaspis sp. G1*
TSA	GAJA	*Leptopilina boulardi*
TSA	GAJC	*Leptopilina heterotoma*
TSA	GAKJ	*Sitodiplosis mosellana*
TSA	GAMD	*Anopheles aquasalis*
TSA	GANH	*Microplitis demolitor*
TSA	GANO	*Corethrella appendiculata*
TSA	GAPE	*Brassicogethes aeneus*
TSA	GAPT	*Ectopsocus briggsi*
TSA	GASE	*Prorhinotermes simplex*
TSA	GASG	*Yponomeuta evonymellus*
TSA	GASN	*Thermobia domestica*
TSA	GASO	*Tricholepidion gertschi*
TSA	GASQ	*Tetrix subulata*
TSA	GASS	*Platycentropus radiatus*
TSA	GAST	*Polyommatus icarus*
TSA	GASV	*Notostira elongata*
TSA	GASW	*Mantis religiosa*
TSA	GASX	*Folsomia candida*
TSA	GASY	*Dyseriocrania subpurpurella*
TSA	GATA	*Meloe violaceus*
TSA	GATB	*Metallyticus splendidus*
TSA	GATC	*Nemophora degeerella*

TSA	GATD	*Pogonognathellus sp. AD-2013*
TSA	GATG	*Corydalus cornutus*
TSA	GATH	*Bittacus pilicornis*
TSA	GATI	*Bombylius major*
TSA	GATJ	*Bibio marci*
TSA	GATU	*Baetis sp. AD-2013*
TSA	GATV	*Perla marginata*
TSA	GATW	*Aleochara curtula*
TSA	GATY	*Chrysis viridula*
TSA	GATZ	*Sminthurus viridis*
TSA	GAUE	*Anurida maritima*
TSA	GAUF	*Leuctra sp. AD-2013*
TSA	GAUG	*Meinertellus cundinamarcensis*
TSA	GAUH	*Panorpa vulgaris*
TSA	GAUI	*Subilla sp. AD-2014*
TSA	GAUM	*Machilis hrabei*
TSA	GAUN	*Cercopis vulnerata*
TSA	GAUO	*Velia caprai*
TSA	GAUV	*Acanthosoma haemorrhoidale*
TSA	GAUW	*Apachyus charteceus*
TSA	GAUX	*Ceuthophilus sp. AD-2013*
TSA	GAUY	*Gyrinus marinus*
TSA	GAUZ	*Stenobothrus lineatus*
TSA	GAVA	*Triarthria setipennis*
TSA	GAVB	*Triodia sylvina*
TSA	GAVM	*Hydroptila sp. AD-2013*
TSA	GAVV	*Pseudomallada prasinus*
TSA	GAVW	*Epiophlebia superstes*
TSA	GAVX	*Timema cristinae*
TSA	GAWC	*Aretaon asperrimus*
TSA	GAWD	*Medauroidea extradentata*
TSA	GAWE	*Ramulus artemis*
TSA	GAWF	*Sipyloidea sipylus*
TSA	GAWG	*Extatosoma tiaratum*
TSA	GAWK	*Ceratophyllus gallinae*
TSA	GAWM	*Culicoides sonorensis*
TSA	GAWP	*Grylloblatta bifratrilecta*
TSA	GAWQ	*Okanagana villosa*
TSA	GAWR	*Menopon gallinae*
TSA	GAWS	*Periplaneta americana*
TSA	GAWT	*Empusa pennata*
TSA	GAWU	*Aposthonia japonica*

TSA	GAWW	*Tenthredo koehleri*
TSA	GAWX	*Trialeurodes vaporariorum*
TSA	GAWZ	*Gryllotalpa sp. AD-2013*
TSA	GAXA	*Isonychia bicolor*
TSA	GAXB	*Tanzaniophasma sp. AD-2013*
TSA	GAXC	*Thrips palmi*
TSA	GAXE	*Acerentomon sp. AD-2013*
TSA	GAXF	*Planococcus citri*
TSA	GAXG	*Gynaikothrips ficorum*
TSA	GAXH	*Parides eurimedes*
TSA	GAXI	*Tetrodontophora bielanensis*
TSA	GAXM	*Mischocyttarus flavitarsis*
TSA	GAXO	*Argochrysis armilla*
TSA	GAXS	*Pepsis grossa*
TSA	GAXT	*Crioscolia alcione*
TSA	GAXW	*Euroleon nostras*
TSA	GAXX	*Rhyacophila fasciata*
TSA	GAXZ	*Trichocera saltator*
TSA	GAYA	*Zorotypus caudelli*
TSA	GAYB	*Zygaena fausta*
TSA	GAYC	*Osmylus fulvicephalus*
TSA	GAYD	*Blaberus atropos*
TSA	GAYE	*Frankliniella cephalica*
TSA	GAYH	*Conwentzia psociformis*
TSA	GAYI	*Xenophysella greensladeae*
TSA	GAYJ	*Atelura formicaria*
TSA	GAYK	*Boreus hyemalis*
TSA	GAYL	*Cosmioperla kuna*
TSA	GAYM	*Calopteryx splendens*
TSA	GAYN	*Campodea augens*
TSA	GAYO	*Cordulegaster boltonii*
TSA	GAYP	*Ctenocephalides felis*
TSA	GAYQ	*Forficula auricularia*
TSA	GAYV	*Liposcelis bostrychophila*
TSA	GAYY	*Acanthocasuarina muellerianae*
TSA	GAYZ	*Ranatra linearis*
TSA	GAZA	*Haploembia palaui*
TSA	GAZB	*Lepicerus sp. AD-2013*
TSA	GAZD	*Lipara lucens*
TSA	GAZE	*Mastotermes darwiniensis*
TSA	GAZF	*Essigella californica*
TSA	GAZG	*Eurylophella sp. AD-2013*

TSA	GAZH	*Inocellia crassicornis*
TSA	GAZM	*Stylops melittae*
TSA	GAZN	*Cryptocercus wrighti*
TSA	GAZQ	*Aretaon asperrimus*
TSA	GAZT	*Prosarthria teretrirostris*
TSA	GBAB	*Musca domestica*
TSA	GBBP	*Teleopsis dalmanni*
TSA	GBCX	*Dastarcus helophoroides*
TSA	GBDM	*Helicoverpa armigera*
TSA	GBGV	*Polistes metricus*
1KITE	INSnfrTAJRAAPEI	*Machilis hrabei*
1KITE	INSnfrTAFRAAPEI	*Meinertellus cundinamarcensis*
1KITE	INSfrgTAVRAAPEI	*Blaberus atropos*
1KITE	INSfrgTAARAAPEI	*Periplaneta americana*
1KITE	INSytvTCDRAAPEI	*Cryptocercus wrighti*
1KITE	INSbttTCRAAPEI	*Arrhenodes minutus*
1KITE	INSnfrTBERAAPEI	*Gyrinus marinus*
1KITE	INSytvTAJRAAPEI	*Lepicerus sp.*
1KITE	INShauTAYRAAPEI	*Meloe violaceus*
1KITE	INShauTBERAAPEI	*Aleochara curtula*
1KITE	INSbttTIRAAPEI	*Folsomia candida*
1KITE	INSnfrTAIRAAPEI	*Anurida maritima*
1KITE	INSjdsTAKRAAPEI	*Tetrodontophora bielanensis*
1KITE	INShauTAFRAAPEI	*Sminthurus viridis/nigromaculatus*
1KITE	INShauTAJRAAPEI	*Pogonognathellus longicornis/flavescens*
1KITE	INSfrgTALRAAPEI	*Apachyus charteceus*
1KITE	INSjdsTBNRAAPEI	*Forficula auricularia*
1KITE	INStmbTABRAAPEI	*Campodea augens*
1KITE	INSjdsTANRAAPEI	*Occasjapyx japonicus*
1KITE	INSbusTBKRAAPEI	*Bombylius major*
1KITE	INSytvTBWRAAPEI	*Lipara lucens*
1KITE	INSnfrTBFRAAPEI	*Triarthria setipennis*
1KITE	INSbusTBCRABPEI	*Bibio marci*
1KITE	INSjdsTBERAAPEI	*Trichocera saltator*
1KITE	INSfrgTAZRAAPEI	*Aposthonia japonica*
1KITE	INSytvTAHRAAPEI	*Haploembia palaui*
1KITE	INShauTAKRAAPEI	*Baetis sp.*
1KITE	INSytvTCERAAPEI	*Eurylophella sp.*
1KITE	INSnfrTAKRAAPEI	*Ephemera danica*
1KITE	INSjdsTAGRAAPEI	*Isonychia bicolor*
1KITE	INSfrgTAKRAAPEI	*Galloisiana yuasai*
1KITE	INSnfrTBKRAAPEI	*Grylloblatta bifratrilecta*

1KITE	INSnfrTANRAAPEI	*Cercopis vulnerata*
1KITE	INSnfrTBLRAAPEI	*Okanagana villosa*
1KITE	INSfrgTBCRAAPEI	*Nilaparvata lugens*
1KITE	INSjdsTARRAAPEI	*Xenophysella greensladeae*
1KITE	INSnfrTAPRAAPEI	*Acanthosoma haemorrhoidale*
1KITE	INShauTAPRAAPEI	*Notostira elongata*
1KITE	INSytvTANRAAPEI	*Ranatra linearis*
1KITE	INSnfrTAORAAPEI	*Velia caprai*
1KITE	INSfrgTAPRAAPEI	*Trialeurodes vaporariorum*
1KITE	INSytvTBHRAAPEI	*Essigella californica*
1KITE	INSjdsTAIRAAPEI	*Planococcus citri*
1KITE	INSytvTALRAAPEI	*Acanthocasuarina muellerianae*
1KITE	INSnfrTAQRAAPEI	*Cotesia vestalis*
1KITE	INShauTAQRABPEI	*Chrysis viridula*
1KITE	INSjdsTAURAAPEI	*Leptopilina clavipes*
1KITE	INSnfrTAARAAPEI	*Orussus abietinus*
1KITE	INSfrgTATRAAPEI	*Tenthredo koehleri*
1KITE	INStmbTBPRAAPEI	*Mastotermes darwiniensis*
1KITE	INSbusTBMRAAPEI	*Prorhinotermes simplex*
1KITE	INShauTABRAAPEI	*Nemophora degeerella*
1KITE	INSbusTBDRAAPEI	*Dyseriocrania subpurpurella*
1KITE	INSnfrTAVRAAPEI	*Triodia sylvina*
1KITE	INShauTBGRAAPEI	*Polyommatus icarus*
1KITE	INSjdsTAJRAAPEI	*Parides eurimedes*
1KITE	INShauTBFRAAPEI	*Yponomeuta evonymellus*
1KITE	INSjdsTAWRAAPEI	*Zygaena fausta*
1KITE	INSfrgTASRAAPEI	*Empusa pennata*
1KITE	INShauTAARAAPEI	*Mantis religiosa*
1KITE	INShauTAMRAAPEI	*Metallyticus splendidus*
1KITE	INSfrgTBBRAAPEI	*Tanzaniophasma sp.*
1KITE	INSbttTARAAPEI	*Bittacus pilicornis*
1KITE	INStmbTAWRAAPEI	*Boreus hyemalis*
1KITE	INShauTACRAAPEI	*Panorpa vulgaris*
1KITE	INSbttTKRAAPEI	*Corydalus cornutus*
1KITE	INSnfrTARRAAPEI	*Pseudomallada prasinus*
1KITE	INSjdsTBQRAAPEI	*Conwentzia psociformis*
1KITE	INSjdsTATRAAPEI	*Euroleon nostras*
1KITE	INSjdsTBJRAAPEI	*Osmylus fulvicephalus*
1KITE	INSjdsTBHRAAPEI	*Cordulegaster boltonii*
1KITE	INSfrgTAHRAAPEI	*Epiophlebia superstes*
1KITE	INStmbTAARAAPEI	*Calopteryx splendens*
1KITE	INSnfrTAMRAAPEI	*Stenobothrus lineatus*

1KITE	INStmbTBCRBAPEI	*Prosarthria teretrirostris*
1KITE	INShauTANRAAPEI	*Tetrix subulata*
1KITE	INSfrgTAXRABPEI	*Gryllotalpa sp.*
1KITE	INSnfrTBIRAAPEI	*Ceuthophilus sp.*
1KITE	INStmbTBERAAPEI	*Aretaon asperrimus*
1KITE	INSfrgTAORAAPEI	*Peruphasma schultei*
1KITE	INSnfrTBPRAAPEI	*Timema cristinae*
1KITE	INStmbTBFRAAPEI	*Cosmioperla kuna*
1KITE	INSnfrTALRAAPEI	*Leuctra sp.*
1KITE	INShauTALRAAPEI	*Perla marginata*
1KITE	INSjdsTAHRAAPEI	*Acerentomon sp.*
1KITE	INSfrgTAFRAAPEI	*Menopon gallinae*
1KITE	INSytvTCFRAAPEI	*Ectopsocus briggsi*
1KITE	INStmbTBGRAAPEI	*Liposcelis bostrychophila*
1KITE	INSnfrTAGRAAPEI	*Xanthostigma xanthostigma*
1KITE	INSnfrTBARAAPEI	*Ceratophyllus gallinae*
1KITE	INStmbTAYRAAPEI	*Ctenocephalides felis*
1KITE	INSytvTBKRAAPEI	*Stylops melittae*
1KITE	INSjdsTADRAAPEI	*Gynaikothrips ficorum*
1KITE	INSjdsTABRAAPEI	*Frankliniella cephalica*
1KITE	INSjdsTACRAAPEI	*Thrips palmi*
1KITE	INSnfrTBJRAAPEI	*Hydroptila actia/argosa*
1KITE	INSjdsTBSRAAPEI	*Rhyacophila fasciata*
1KITE	INSjdsTAQRAAPEI	*Zorotypus caudelli*
1KITE	INSjdsTAVRAAPEI	*Atelura formicaria*
1KITE	INSbttTJRAAPEI	*Tricholepidion gertschi*
1KITE	INSbttTSRAAPEI	*Thermobia domestica*

Supplementary File 1B: List of *oskar* sequences used in the final alignment.
List of accession numbers and database provenance of the sequences used in the final alignments of Oskar analysed herein. The table contains the database provenance (*Type*), the database accession number (*ID*), the species, family and order, and extraction notes. In the "Annotation" Column, P = homolog identified by pipeline; DB = homolog identified by database annotation. *Sequence recomposed from two transcripts: GBCX01024638.1 and GBCX01024637.

Type	ID	*Species*	Family	Order	Annotation	Note
TSA	GAWC01068734.1	*Aretaon asperrimus*	Heteropterygidae	Phasmatodea	P	HMMER
TSA	GAKJ01010751.1	*Sitodiplosis mosellana*	Cecidomyiidae	Diptera	P	HMMER
TSA	GATI01010233.1	*Bombylius major*	Bombyliidae	Diptera	P	HMMER
TSA	GAWW01000144.1	*Tenthredo koehleri*	Tenthredinidae	Hymenoptera	P	HMMER
TSA	GAIW01009539.1	*Ganaspis sp.*	Figitidae	Hymenoptera	P	HMMER
TSA	GATY01008637.1	*Chrysis viridula*	Chrysididae	Hymenoptera	P	HMMER
TSA	GAXM01030263.1	*Mischocyttarus flavitarsis*	Vespidae	Hymenoptera	P	HMMER
TSA	GAFR01040300.1	*Polistes canadensis*	Vespidae	Hymenoptera	P	HMMER
TSA	GAZD01106195.1	*Lipara lucens*	Chloropidae	Diptera	P	HMMER
TSA	GAVA01002196.1	*Triarthria setipennis*	Tachinidae	Diptera	P	HMMER
TSA	GACV01001831.1	*Philopotamus ludificatus*	Philopotaminae	Trichoptera	P	HMMER
TSA	GAPE01019095.1	*Brassicogethes aeneus*	Nitidulidae	Coleoptera	P	HMMER
TSA	GBCX01024638.1_7.1	*Dastarcus helophoroides**	Bothrideridae	Coleoptera	P	BLAST 1
TSA	GAKJ01010751.1	*Sitodiplosis mosellana*	Cecidomyiidae	Diptera	P	HMMER
TSA	GAWM01006639.1	*Culicoides sonorensis*	Ceratopogonidae	Diptera	P	HMMER
TSA	GAMD01000859.1	*Anopheles aquasalis*	Culicidae	Diptera	P	HMMER
TSA	GBEO01001325.1	*Anopheles sinensis*	Culicidae	Diptera	P	HMMER
TSA	GAKP01002609.1	*Bactrocera dorsalis*	Tephritidae	Diptera	P	HMMER
TSA	GAXM01030263.1	*Mischocyttarus flavitarsis*	Vespidae	Hymenoptera	P	HMMER
TSA	GAFR01040300.1	*Polistes canadensis canadensis*	Vespidae	Hymenoptera	P	HMMER
TSA	GBGV01010610.1	*Polistes metricus*	Vespidae	Hymenoptera	P	HMMER
TSA	GAIW01011550.1	*Ganaspis sp. G1*	Figitidae	Hymenoptera	P	HMMER
TSA	GAJC01011221.1	*Leptopilina heterotoma*	Figitidae	Hymenoptera	P	HMMER
TSA	GAIW01009539.1	*Ganaspis sp. G1*	Figitidae	Hymenoptera	P	HMMER

TSA	GAJA01020544.1	*Leptopilina boulardi*	Figitidae	Hymenoptera	P	HMMER
TSA	GAJC01009625.1	*Leptopilina heterotoma*	Figitidae	Hymenoptera	P	HMMER
TSA	GAXO01016630.1	*Argochrysis armilla*	Chrysididae	Hymenoptera	P	HMMER
TSA	GAEO01004319.1	*Pissodes strobi*	Curculionidae	Coleoptera	P	HMMER
TSA	GBBP01080309.1	*Teleopsis dalmanni*	Diopsidae	Diptera	P	HMMER
TSA	GAVM01000124.1	*Hydroptila actia/argosa*	Hydroptilidae	Trichoptera	P	HMMER
TSA	GAWC01068728	*Aretaon asperrimus*	Heteropterygidae	Phasmatodea	P	HMMER
TSA	GBEO01001325.1	*Anopheles sinensis*	Culicidae	Diptera	P	HMMER
Genome	GCA_000349125.1	*Anopheles albimanus*	Culicidae	Diptera	P	Snap
Genome	GCA_000349065.1	*Anopheles quadriannulatus*	Culicidae	Diptera	P	Augustus
Genome	GCA_000349185.1	*Anopheles arabiensis*	Culicidae	Diptera	P	Augustus
Genome	GCA_000473845.2	*Anopheles merus*	Culicidae	Diptera	P	Augustus
Genome	GCA_000473375.1	*Anopheles culicifacies*	Culicidae	Diptera	P	Augustus
Genome	GCA_000349085.1	*Anopheles funestus*	Culicidae	Diptera	P	Augustus
Genome	GCA_000209185.1	*Culex quinquefasciatus*	Culicidae	Diptera	P	Snap
Genome	GCF_000004015.3	*Aedes aegypti*	Culicidae	Diptera	P	Augustus
Genome	GCA_001014445.1	*Scaptodrosophila lebanonensis*	Drosophilidae	Diptera	P	Snap
Genome	GCA_000005925.1	*Drosophila willistoni*	Drosophilidae	Diptera	P	Augustus
Genome	GCA_000005115.1	*Drosophila ananassae*	Drosophilidae	Diptera	P	Snap
Genome	GCA_000236285.2	*Drosophila bipectinata*	Drosophilidae	Diptera	P	Augustus
Genome	GCA_000224215.2	*Drosophila kikkawai*	Drosophilidae	Diptera	P	Snap
Genome	GCA_000236325.2	*Drosophila eugracilis*	Drosophilidae	Diptera	P	Augustus
Genome	GCA_000001215.4	*Drosophila melanogaster*	Drosophilidae	Diptera	P	Augustus
Genome	GCA_000259055.1	*Drosophila simulans*	Drosophilidae	Diptera	P	Snap
Genome	GCA_000005215.1	*Drosophila sechellia*	Drosophilidae	Diptera	P	Augustus
Genome	GCA_000005135.1	*Drosophila erecta*	Drosophilidae	Diptera	P	Augustus
Genome	GCA_000005975.1	*Drosophila yakuba*	Drosophilidae	Diptera	P	Augustus
Genome	GCA_000220665.2	*Drosophila ficusphila*	Drosophilidae	Diptera	P	Snap
Genome	GCA_000224195.2	*Drosophila elegans*	Drosophilidae	Diptera	P	Snap
Genome	GCA_000236305.2	*Drosophila rhopaloa*	Drosophilidae	Diptera	P	Augustus
Genome	GCA_000233415.2	*Drosophila biarmipes*	Drosophilidae	Diptera	P	Snap

Genome	GCA_000224235.2	*Drosophila takahashii*	Drosophilidae	Diptera	P	Augustus
Genome	GCA_000269505.2	*Drosophila miranda*	Drosophilidae	Diptera	P	Augustus
Genome	GCA_000001765.2	*Drosophila pseudoobscura*	Drosophilidae	Diptera	P	Snap
Genome	GCA_000005195.1	*Drosophila persimilis*	Drosophilidae	Diptera	P	Snap
Genome	GCA_000298335.1	*Drosophila albomicans*	Drosophilidae	Diptera	P	Snap
Genome	GCA_000005155.1	*Drosophila grimshawi*	Drosophilidae	Diptera	P	Snap
Genome	GCA_000005175.1	*Drosophila mojavensis*	Drosophilidae	Diptera	P	Snap
Genome	GCA_000005245.1	*Drosophila virilis*	Drosophilidae	Diptera	P	Snap
Genome	GCA_000371365.1	*Musca domestica*	Muscidae	Diptera	P	Augustus
Genome	GCA_000648655.1	*Copidosoma floridanum*	Encyrtidae	Hymenoptera	P	Blast
Protein	ABC54566.1	*Anopheles gambiae*	Culicidae	Diptera	DB	-
Protein	KYN09041	*Trachymyrmex cornetzi*	Formicidae	Hymenoptera	DB	-
Protein	KYM88541	*Atta colombica*	Formicidae	Hymenoptera	DB	-
Protein	XP_012060266	*Atta cephalotes*	Formicidae	Hymenoptera	DB	-
Protein	XP_011057669	*Acromyrmex echinatior*	Formicidae	Hymenoptera	DB	-
Protein	ADM07366	*Messor pergandei*	Formicidae	Hymenoptera	DB	-
Protein	XP_008556449	*Microplitis demolitor*	Microgastrinae	Hymenoptera	DB	-
Protein	XP_012229836	*Linepithema humile*	Dolichoderinae	Hymenoptera	DB	-
Protein	ABC54566	*Anopheles gambiae*	Culicidae	Diptera	DB	-
Protein	ACB20969	*Culex quinquefasciatus*	Culicidae	Diptera	DB	-
Protein	ABC41128	*Aedes aegypti*	Culicidae	Diptera	DB	-
Protein	XP_004529162	*Ceratitis capitata*	Tephritidae	Diptera	DB	-
Protein	NP_001234884	*Nasonia vitripennis*	Pteromalidae	Hymenoptera	DB	-
Protein	XM_011167600	*Solenopsis invicta*	Formicidae	Hymenoptera	DB	-
Protein	JR477371.1	*Rhynchophorus errugineus*	Curculionidae	Coleoptera	DB	-
Nucleotide	JO902149	*Aedes albopictus*	Aedes	Diptera	DB	-
Nucleotide	JO874052	*Aedes albopictus*	Aedes	Diptera	DB	-
Nucleotide	JO885398	*Aedes albopictus*	Aedes	Diptera	DB	-
1KITE	INStmbTBGRAAPEI-33	*Liposcelis bostrychophila*	Liposcelididae	Psocodea	P	Blast
1KITE	INSjdsTABRAAPEI-20	*Frankliniella cephalica*	Thripidae	Thysanoptera	P	Blast
1KITE	INSjdsTACRAAPEI-21	*Thrips palmi*	Thripidae	Thysanoptera	P	Blast

1KITE	INShauTABRAAPEI-93	*Nemophora degeerella*	Adelidae	Lepidoptera	P	Blast
1KITE	INSbttTARAAPEI-9	*Platycentropus radiatus*	Limnephilidae	Trichoptera	P	Blast
1KITE	INSnfrTALRAAPEI-31	*Leuctra sp.*	Leuctridae	Plecoptera	P	Blast
1KITE	INShauTAKRAAPEI-90	*Baetis pumilus*	Baetidae	Ephemeroptera	P	Blast
1KITE	INSjdsTADRAAPEI-22	*Gynaikothrips ficorum*	Phlaeothripidae	Thysanoptera	P	Blast
1KITE	INStmbTAWRAAPEI-13	*Boreus hyemalis*	Boreidae	Mecoptera	P	Blast
1KITE	INSytvTCDRAAPEI-35	*Cryptocercus wrighti*	Cryptocercidae	Blattodea	P	Blast
1KITE	INSnfrTAQRAAPEI-37	*Cotesia vestalis*	Braconidae	Hymenoptera	P	Blast
1KITE	INSjdsTAURAAPEI-62	*Leptopilina clavipes*	Figitidae	Hymenoptera	P	Blast

Supplementary File 1C: List of sequences and their BLAST results used for phylogenetic analysis of the LOTUS domain. The sequences were obtained by searching the TrEMBL database using hmmsearch and the final HMM generated for LOTUS (Supplementary files: HMM>LOTUS.hmm). Reported are the UniProtID (*Accession Number*), the Domain and Phylum origin of the sequence, the E-value, score and bias given by hmmsearch, and the description of the target from UniProt. To obtain sequences for each entry, either search UniProt directly (https://www.uniprot.org/) or consult the final alignment in Supplementary Files: Alignments>LOTUS_TREE.fasta. Phylum abbreviations: A = Arthropoda; An = Annelida; E = Echinodermata; F = Firmicutes; M = Mollusca; T = Tunicata; V = Vertebrata; ? = unclassified

Accession ID	Domain	Phylum	E-value	score	bias	Description of Target
V3Z0B0_LOTGI	Eukarya	M	8.30E-32	120.8	0.1	Uncharacterized protein OS=Lottia gigantea GN=LOTGIDRAFT_236389 PE=4 SV=1
R7UJX3_CAPTE	Eukarya	An	4.00E-30	115.3	0	Uncharacterized protein OS=Capitella teleta GN=CAPTEDRAFT_218952 PE=4 SV=1
E9IZ46_SOLIN	Eukarya	A	2.80E-27	106.2	0.1	Putative uncharacterized protein (Fragment) OS=Solenopsis invicta GN=SINV_01516 PE=4 SV=1
K7JUZ2_NASVI	Eukarya	A	9.00E-27	104.6	0.2	Uncharacterized protein OS=Nasonia vitripennis GN=oskar PE=4 SV=1
F4WQN7_ACREC	Eukarya	A	1.30E-25	100.9	0.1	Maternal effect protein oskar OS=Acromyrmex echinatior GN=G5I_08127 PE=4 SV=1
W4ZBK4_STRPU	Eukarya	E	1.70E-25	100.5	0.1	Uncharacterized protein OS=Strongylocentrotus purpuratus GN=Sp-Tdrd5 PE=4 SV=1
E2A7I8_CAMFO	Eukarya	A	1.90E-25	100.4	2.1	Putative uncharacterized protein OS=Camponotus floridanus GN=EAG_03874 PE=4 SV=1
F2WJY6_9HYME	Eukarya	A	3.40E-25	99.6	0	Oskar (Fragment) OS=Messor pergandei PE=2 SV=1
B4N816_DROWI	Eukarya	A	8.50E-25	98.3	9.6	Uncharacterized protein OS=Drosophila willistoni GN=Dwil\GK11116 PE=4 SV=2
B4K9E5_DROMO	Eukarya	A	8.70E-25	98.2	0.6	Uncharacterized protein OS=Drosophila mojavensis GN=Dmoj\GI10055 PE=4 SV=2
A0A026WMY1_CERBI	Eukarya	A	2.20E-24	97	0.1	Maternal effect protein oskar OS=Cerapachys biroi GN=X777_01612 PE=4 SV=1
C3ZCL9_BRAFL	Eukarya	A	5.40E-24	95.7	0	Putative uncharacterized protein OS=Branchiostoma floridae GN=BRAFLDRAFT_64001 PE=4 SV=1
B4LXK5_DROVI	Eukarya	A	8.00E-24	95.2	0.9	Oskar OS=Drosophila virilis GN=osk PE=4 SV=1
K4MTL4_GRYBI	Eukarya	A	6.70E-23	92.2	0	Oskar OS=Gryllus bimaculatus PE=2 SV=1
B4JTJ1_DROGR	Eukarya	A	8.30E-23	91.9	1.4	GH23955 OS=Drosophila grimshawi GN=Dgri\GH23955 PE=4 SV=1
A1Y1T7_DROIM	Eukarya	A	8.60E-23	91.9	0.8	Oskar OS=Drosophila immigrans GN=osk PE=4 SV=1
Q2PP79_AEDAE	Eukarya	A	1.70E-22	90.9	0	Oskar OS=Aedes aegypti PE=4 SV=1

W8CE30_CERCA	Eukarya	A	2.00E-22	90.7	0.3	Maternal effect protein oskar OS=Ceratitis capitata GN=OSKA PE=2 SV=1
T1PG45_MUSDO	Eukarya	A	1.60E-21	87.8	1.2	GDSL-like Lipase/Acylhydrolase OS=Musca domestica PE=2 SV=1
A0A0L8HW18_OCTBM	Eukarya	M	1.70E-21	87.7	7.5	Uncharacterized protein OS=Octopus bimaculoides GN=OCBIM_22005378mg PE=4 SV=1
F6QYS5_XENTR	Eukarya	V	6.10E-20	82.7	0.2	Uncharacterized protein (Fragment) OS=Xenopus tropicalis PE=4 SV=1
B0WIV7_CULQU	Eukarya	A	8.90E-20	82.2	0.1	Oskar OS=Culex quinquefasciatus GN=CpipJ_CPIJ007471 PE=2 SV=1
A0A034WRF5_BACDO	Eukarya	A	1.30E-19	81.6	13.1	Maternal effect protein oskar OS=Bactrocera dorsalis GN=OSKA PE=4 SV=1
Q7PQJ3_ANOGA	Eukarya	A	1.40E-19	81.5	0	AGAP003545-PA OS=Anopheles gambiae GN=osk PE=4 SV=3
X1WIW2_ACYPI	Eukarya	A	1.70E-19	81.3	12.3	Uncharacterized protein OS=Acyrthosiphon pisum GN=LOC100162069 PE=4 SV=1
A0A023EWV9_TRIIF	Eukarya	A	1.80E-19	81.2	0.2	Putative transcriptional coactivator (Fragment) OS=Triatoma infestans PE=2 SV=1
A0A0L0CP24_LUCCU	Eukarya	A	2.40E-19	80.8	0.5	Uncharacterized protein OS=Lucilia cuprina GN=FF38_12727 PE=4 SV=1
A0A0K8W0W3_BACLA	Eukarya	A	5.00E-19	79.8	0	Maternal effect protein oskar OS=Bactrocera latifrons GN=osk_1 PE=4 SV=1
A0A0A1XRQ4_BACCU	Eukarya	A	7.20E-19	79.3	6.4	Maternal effect protein oskar OS=Bactrocera cucurbitae GN=osk PE=4 SV=1
A0A088ASD6_APIME	Eukarya	A	2.90E-18	77.3	0.2	Uncharacterized protein OS=Apis mellifera GN=LOC726241 PE=4 SV=1
K1QZD2_CRAGI	Eukarya	M	1.60E-17	75	0	Tudor domain-containing protein 7 OS=Crassostrea gigas GN=CGI_10018436 PE=4 SV=1
A0A139WGI6_TRICA	Eukarya	A	1.70E-17	74.9	0.1	Tudor domain-containing protein 7-like protein OS=Tribolium castaneum GN=TcasGA2_TC034722 PE=4 SV=1
F6YH90_ORNAN	Eukarya	V	1.80E-17	74.8	0	Uncharacterized protein OS=Ornithorhynchus anatinus GN=TDRD5 PE=4 SV=1
W5JJ85_ANODA	Eukarya	A	2.10E-17	74.6	0	Uncharacterized protein OS=Anopheles darlingi GN=AND_005442 PE=4 SV=1
A0A084WRU4_ANOSI	Eukarya	A	2.40E-17	74.4	0.2	AGAP003545-PA-like protein OS=Anopheles sinensis GN=ZHAS_00021239 PE=4 SV=1
H2YLA8_CIOSA	Eukarya	T	2.60E-17	74.3	0.1	Uncharacterized protein OS=Ciona savignyi GN=Csa.10307 PE=4 SV=1
G1KVT0_ANOCA	Eukarya	V	2.70E-17	74.2	0.1	Uncharacterized protein OS=Anolis carolinensis GN=TDRD5 PE=4 SV=1
G3TEV7_LOXAF	Eukarya	V	4.50E-17	73.5	0	Uncharacterized protein OS=Loxodonta africana GN=TDRD5 PE=4 SV=1
A0A0K8TEH4_LYGHE	Eukarya	A	6.40E-17	73	0	Uncharacterized protein OS=Lygus hesperus PE=4 SV=1

A0A067RPA3_ZOONE	Eukarya	A	7.10E-17	72.9	0.1	Tudor domain-containing protein 7 OS=Zootermopsis nevadensis GN=L798_01728 PE=4 SV=1
G3VEY7_SARHA	Eukarya	V	1.30E-16	72.1	0	Uncharacterized protein OS=Sarcophilus harrisii GN=TDRD5 PE=4 SV=1
L8I7L7_9CETA	Eukarya	V	1.40E-16	72	0.1	Tudor domain-containing protein 5 (Fragment) OS=Bos mutus GN=M91_03486 PE=4 SV=1
W5Q779_SHEEP	Eukarya	V	1.40E-16	71.9	0.5	Uncharacterized protein OS=Ovis aries GN=TDRD5 PE=4 SV=1
G5E528_BOVIN	Eukarya	V	2.30E-16	71.3	0	Tudor domain-containing protein 5 OS=Bos taurus GN=TDRD5 PE=4 SV=1
H2MII4_ORYLA	Eukarya	V	2.90E-16	71	0	Uncharacterized protein OS=Oryzias latipes GN=TDRD7 (1 to many) PE=4 SV=1
N6TQX5_DENPD	Eukarya	A	3.20E-16	70.8	0.1	Uncharacterized protein (Fragment) OS=Dendroctonus ponderosae GN=YQE_11709 PE=4 SV=1
F6WY93_HORSE	Eukarya	V	5.80E-16	70	0	Uncharacterized protein OS=Equus caballus GN=TDRD5 PE=4 SV=1
A0A0J9YJ00_DANRE	Eukarya	V	5.90E-16	69.9	0	Tudor domain-containing protein 7A (Fragment) OS=Danio rerio GN=tdrd7a PE=1 SV=2
U5EFJ8_9DIPT	Eukarya	A	9.50E-16	69.3	0.9	Putative oskar (Fragment) OS=Corethrella appendiculata PE=2 SV=1
H2USX7_TAKRU	Eukarya	V	9.60E-16	69.3	0	Uncharacterized protein OS=Takifugu rubripes PE=4 SV=1
F1S6A1_PIG	Eukarya	V	1.10E-15	69.1	0	Uncharacterized protein OS=Sus scrofa GN=TDRD5 PE=4 SV=2
T1DTM7_ANOAQ	Eukarya	A	1.10E-15	69	0	Uncharacterized protein (Fragment) OS=Anopheles aquasalis PE=2 SV=1
A0A060W2X9_ONCMY	Eukarya	V	2.20E-15	68.1	0	Uncharacterized protein OS=Oncorhynchus mykiss GN=GSONMT00078733001 PE=4 SV=1
S7NG41_MYOBR	Eukarya	V	2.60E-15	67.9	0.1	Tudor domain-containing protein 5 OS=Myotis brandtii GN=D623_10022817 PE=4 SV=1
F7B4W0_MONDO	Eukarya	V	2.70E-15	67.8	0	Uncharacterized protein OS=Monodelphis domestica GN=TDRD5 PE=4 SV=2
V9KH94_CALMI	Eukarya	V	3.50E-15	67.5	0	Tudor domain-containing protein 5 OS=Callorhinchus milii PE=2 SV=1
A0A0P7YHR6_9TELE	Eukarya	V	4.70E-15	67	0	Uncharacterized protein OS=Scleropages formosus GN=Z043_114704 PE=4 SV=1
G1PFT9_MYOLU	Eukarya	V	5.70E-15	66.8	0.1	Uncharacterized protein OS=Myotis lucifugus GN=TDRD5 PE=4 SV=1
H3DL34_TETNG	Eukarya	V	7.70E-15	66.4	0	Uncharacterized protein OS=Tetraodon nigroviridis PE=4 SV=1
A0A096NXU4_PAPAN	Eukarya	V	8.70E-15	66.2	0	Uncharacterized protein OS=Papio anubis GN=TDRD5 PE=4 SV=1
F7GPY1_CALJA	Eukarya	V	1.10E-14	65.9	0	Uncharacterized protein OS=Callithrix jacchus GN=TDRD5 PE=4 SV=1

W5N030_LEPOC	Eukarya	V	1.20E-14	65.8	0.1	Uncharacterized protein OS=Lepisosteus oculatus PE=4 SV=1
M3VXB3_FELCA	Eukarya	V	1.30E-14	65.6	0	Uncharacterized protein OS=Felis catus GN=TDRD5 PE=4 SV=1
A0A099ZTT8_TINGU	Eukarya	V	1.40E-14	65.6	0	Tudor domain-containing protein 7 OS=Tinamus guttatus GN=N309_12928 PE=4 SV=1
G3R6R4_GORGO	Eukarya	V	1.50E-14	65.5	0	Uncharacterized protein OS=Gorilla gorilla gorilla GN=TDRD5 PE=4 SV=1
L9L889_TUPCH	Eukarya	V	1.60E-14	65.4	0.3	Tudor domain-containing protein 5 OS=Tupaia chinensis GN=TREES_T100015801 PE=4 SV=1
A0A0J7KVQ7_LASNI	Eukarya	A	1.70E-14	65.3	0	Tudor domain-containing protein 5 (Fragment) OS=Lasius niger GN=RF55_5458 PE=4 SV=1
E0VJL4_PEDHC	Eukarya	A	1.70E-14	65.3	0.9	Putative uncharacterized protein OS=Pediculus humanus subsp. corporis GN=Phum_PHUM247930 PE=4 SV=1
A0A0D9RID1_CHLSB	Eukarya	V	1.70E-14	65.2	0	Uncharacterized protein OS=Chlorocebus sabaeus GN=TDRD5 PE=4 SV=1
E2BFZ8_HARSA	Eukarya	A	2.00E-14	65	0.9	Tudor domain-containing protein 7 OS=Harpegnathos saltator GN=EAI_14615 PE=4 SV=1
A0A0T6AU98_9SCAR	Eukarya	A	2.10E-14	65	3.9	Uncharacterized protein (Fragment) OS=Oryctes borbonicus GN=AMK59_7658 PE=4 SV=1
R9PXP1_CHICK	Eukarya	V	2.30E-14	64.8	0	Tudor domain-containing protein 7 OS=Gallus gallus GN=TDRD7 PE=4 SV=1
F7CN93_MACMU	Eukarya	V	2.70E-14	64.6	0	Uncharacterized protein OS=Macaca mulatta GN=TDRD5 PE=4 SV=1
A0A0A0MPC8_CANLF	Eukarya	V	2.70E-14	64.6	0	Tudor domain-containing protein 5 OS=Canis lupus familiaris GN=TDRD5 PE=4 SV=1
M3Y1J3_MUSPF	Eukarya	V	2.90E-14	64.5	0	Uncharacterized protein OS=Mustela putorius furo GN=TDRD5 PE=4 SV=1
H2N4J0_PONAB	Eukarya	V	3.00E-14	64.5	0	Uncharacterized protein OS=Pongo abelii GN=TDRD5 PE=4 SV=1
H2Q0P6_PANTR	Eukarya	V	3.20E-14	64.4	0	Uncharacterized protein OS=Pan troglodytes GN=TDRD5 PE=4 SV=1
W5LEX2_ASTMX	Eukarya	V	3.50E-14	64.3	0	Uncharacterized protein OS=Astyanax mexicanus PE=4 SV=1
A0A024R910_HUMAN	Eukarya	V	4.00E-14	64.1	0	Tudor domain containing 5, isoform CRA_b OS=Homo sapiens GN=TDRD5 PE=4 SV=1
A0A0P6JFX9_HETGA	Eukarya	V	4.10E-14	64.1	0	Tudor domain-containing protein 5 isoform 2 OS=Heterocephalus glaber GN=TDRD5 PE=4 SV=1
H0WXJ6_OTOGA	Eukarya	V	4.70E-14	63.8	0	Uncharacterized protein OS=Otolemur garnettii GN=TDRD5 PE=4 SV=1
H0V001_CAVPO	Eukarya	V	5.20E-14	63.7	0	Uncharacterized protein OS=Cavia porcellus GN=TDRD5 PE=4 SV=1
G7NU06_MACFA	Eukarya	V	5.80E-14	63.6	0	Putative uncharacterized protein OS=Macaca fascicularis GN=EGM_01633 PE=4 SV=1

G1S4T3_NOMLE	Eukarya	V	6.40E-14	63.4	0	Uncharacterized protein OS=Nomascus leucogenys GN=TDRD7 PE=4 SV=1
G1M861_AILME	Eukarya	V	6.80E-14	63.3	0	Tudor domain-containing protein 5 OS=Ailuropoda melanoleuca GN=TDRD5 PE=4 SV=1
A0A061HYN9_CRIGR	Eukarya	V	7.10E-14	63.3	0	Tudor domain-containing protein 5 OS=Cricetulus griseus GN=H671_5g14992 PE=4 SV=1
A0A0F8AWR5_LARCR	Eukarya	V	1.00E-13	62.7	0	Tudor domain-containing protein 7A OS=Larimichthys crocea GN=EH28_10800 PE=4 SV=1
V5GPP4_ANOGL	Eukarya	A	1.10E-13	62.6	1.4	Tudor domain-containing protein (Fragment) OS=Anoplophora glabripennis GN=TDRD7 PE=4 SV=1
A0A087XQP1_POEFO	Eukarya	V	1.10E-13	62.6	0	Uncharacterized protein OS=Poecilia formosa PE=4 SV=2
S4NW46_9NEOP	Eukarya	A	1.20E-13	62.5	0	Tudor domain containing 7 (Fragment) OS=Pararge aegeria PE=4 SV=1
L5KMV4_PTEAL	Eukarya	V	2.10E-13	61.8	0	Tudor domain-containing protein 7 OS=Pteropus alecto GN=PAL_GLEAN10008027 PE=4 SV=1
I3NCP3_ICTTR	Eukarya	V	2.40E-13	61.6	0	Uncharacterized protein OS=Ictidomys tridecemlineatus GN=TDRD7 PE=4 SV=1
G1SCH8_RABIT	Eukarya	V	2.50E-13	61.5	0	Uncharacterized protein OS=Oryctolagus cuniculus GN=TDRD7 PE=4 SV=1
Q3TTK4_MOUSE	Eukarya	V	2.50E-13	61.5	0	Putative uncharacterized protein (Fragment) OS=Mus musculus GN=Tdrd7 PE=2 SV=1
A0A0H2UHC6_RAT	Eukarya	V	3.90E-13	60.9	0	Tudor domain-containing protein 5 OS=Rattus norvegicus GN=Tdrd5 PE=4 SV=1
U6CRG5_NEOVI	Eukarya	V	3.90E-13	60.9	0	Tudor domain-containing protein 7 OS=Neovison vison GN=TDRD7 PE=2 SV=1
K7FVR3_PELSI	Eukarya	V	5.10E-13	60.5	0	Uncharacterized protein OS=Pelodiscus sinensis GN=TDRD5 PE=4 SV=1
J4K9P6_9FIRM	Bacteria	F	2.90E-07	42.1	31.8	NYN domain protein OS=Peptostreptococcaceae bacterium AS15 GN=HMPREF1142_1162 PE=4 SV=1
E0QL58_9FIRM	Bacteria	F	0.00014	33.5	29.1	Uncharacterized protein OS=[Eubacterium] yurii subsp. margaretiae ATCC 43715 GN=HMPREF0379_1756 PE=4 SV=1
A0A0G0IVI6_9BACT	Bacteria	?	0.0025	29.5	1.9	Uncharacterized protein OS=candidate division TM6 bacterium GW2011_GWA2_36_9 GN=US32_C0002G0021 PE=4 SV=1

Supplementary File 1D: List of sequences and their BLAST results used for phylogenetic analysis of the OSK domain. The sequences were obtained by searching the TrEMBL database using hmmsearch and the final HMM generated for OSK (Supplementary files: HMM>OSK.hmm). Reported parameters are as described for Supplementary Table S3. To obtain sequences for each entry, either search UniProt directly (https://www.uniprot.org/) or consult the final alignment in Supplementary Files: Alignments>OSK_TREE.fasta. Phylum Abbreviations: A = Arthropoda; Ar = Archaea; As = Ascomycota; B = Bacteroidetes; C = Cyanobacteria; Eu = Euryarchaeota; F = Firmicutes; Fu = Fungi; P = Proteobacteria

Accession Number	Domain	Phylum	E-value	Score	Bias	Description of target
A1Y1T7_DROIM	Eukarya	A	2.90E-34	128.4	0.6	Oskar OS=Drosophila immigrans GN=osk PE=4 SV=1
F2WJY6_9HYME	Eukarya	A	3.50E-34	128.2	0.9	Oskar (Fragment) OS=Messor pergandei PE=2 SV=1
B4JTJ1_DROGR	Eukarya	A	5.00E-34	127.6	0.1	GH23955 OS=Drosophila grimshawi GN=Dgri\GH23955 PE=4 SV=1
F4WQN7_ACREC	Eukarya	A	8.10E-34	127	0.9	Maternal effect protein oskar OS=Acromyrmex echinatior GN=G5I_08127 PE=4 SV=1
E2A7I8_CAMFO	Eukarya	A	2.80E-33	125.2	0.4	Putative uncharacterized protein OS=Camponotus floridanus GN=EAG_03874 PE=4 SV=1
W8CE30_CERCA	Eukarya	A	5.80E-33	124.2	0.3	Maternal effect protein oskar OS=Ceratitis capitata GN=OSKA PE=2 SV=1
A0A0A1XRQ4_BACCU	Eukarya	A	7.50E-33	123.9	0.4	Maternal effect protein oskar OS=Bactrocera cucurbitae GN=osk PE=4 SV=1
B4N815_DROWI	Eukarya	A	8.10E-33	123.8	0.3	Uncharacterized protein OS=Drosophila willistoni GN=Dwil\GK11117 PE=4 SV=2
A0A026WMY1_CERBI	Eukarya	A	2.40E-32	122.3	0.7	Maternal effect protein oskar OS=Cerapachys biroi GN=X777_01612 PE=4 SV=1
E9IZ46_SOLIN	Eukarya	A	2.50E-32	122.2	0.1	Putative uncharacterized protein (Fragment) OS=Solenopsis invicta GN=SINV_01516 PE=4 SV=1
A0A034WRF5_BACDO	Eukarya	A	2.70E-32	122.1	0.5	Maternal effect protein oskar OS=Bactrocera dorsalis GN=OSKA PE=4 SV=1
A0A0K8U7J3_BACLA	Eukarya	A	5.70E-32	121	0.5	Maternal effect protein oskar OS=Bactrocera latifrons GN=osk_2 PE=4 SV=1
Q2PP79_AEDAE	Eukarya	A	6.30E-32	120.9	0.1	Oskar OS=Aedes aegypti PE=4 SV=1
B4K9E5_DROMO	Eukarya	A	8.60E-32	120.5	0.1	Uncharacterized protein OS=Drosophila mojavensis GN=Dmoj\GI10055 PE=4 SV=2
B4LXK5_DROVI	Eukarya	A	1.20E-31	120	0.1	Oskar OS=Drosophila virilis GN=osk PE=4 SV=1
A0A0M4F3M8_DROBS	Eukarya	A	1.80E-31	119.4	0.4	Osk OS=Drosophila busckii GN=Dbus_chr3Rg607 PE=4 SV=1
A0A0L0CP24_LUCCU	Eukarya	A	2.90E-31	118.8	1.5	Uncharacterized protein OS=Lucilia cuprina GN=FF38_12727 PE=4 SV=1
B4PTX6_DROYA	Eukarya	A	1.00E-30	117	0.5	Uncharacterized protein OS=Drosophila yakuba GN=Dyak\GE25914 PE=4 SV=1

B3P1W4_DROER	Eukarya	A	2.70E-30	115.6	0.5	GG13545 OS=Drosophila erecta GN=Dere\GG13545 PE=4 SV=1
T1PG45_MUSDO	Eukarya	A	4.20E-30	115.1	0.7	GDSL-like Lipase/Acylhydrolase OS=Musca domestica PE=2 SV=1
B4HKZ1_DROSE	Eukarya	A	5.20E-30	114.8	0.2	GM23770 OS=Drosophila sechellia GN=Dsec\GM23770 PE=4 SV=1
Q295Q4_DROPS	Eukarya	A	6.40E-30	114.5	0.2	Uncharacterized protein, isoform A OS=Drosophila pseudoobscura pseudoobscura GN=Dpse\GA10627 PE=4 SV=2
E8NH25_DROME	Eukarya	A	7.30E-30	114.3	0.2	RE24380p (Fragment) OS=Drosophila melanogaster GN=osk-RA PE=2 SV=1
T1DTM7_ANOAQ	Eukarya	A	1.00E-29	113.8	0	Uncharacterized protein (Fragment) OS=Anopheles aquasalis PE=2 SV=1
B3LZ06_DROAN	Eukarya	A	2.20E-28	109.5	0.1	Uncharacterized protein OS=Drosophila ananassae GN=Dana\GF17692 PE=4 SV=1
E1A883_NASVI	Eukarya	A	4.20E-28	108.6	0.2	Oskar OS=Nasonia vitripennis PE=2 SV=1
A0A084WRU4_ANOSI	Eukarya	A	1.30E-27	107.1	0.1	AGAP003545-PA-like protein OS=Anopheles sinensis GN=ZHAS_00021239 PE=4 SV=1
W5JJ85_ANODA	Eukarya	A	4.50E-27	105.3	0	Uncharacterized protein OS=Anopheles darlingi GN=AND_005442 PE=4 SV=1
Q7PQJ3_ANOGA	Eukarya	A	6.00E-27	104.9	0	AGAP003545-PA OS=Anopheles gambiae GN=osk PE=4 SV=3
B0WIV7_CULQU	Eukarya	A	1.30E-26	103.9	0.4	Oskar OS=Culex quinquefasciatus GN=CpipJ_CPIJ007471 PE=2 SV=1
U5EFJ8_9DIPT	Eukarya	A	4.00E-25	99.1	0	Putative oskar (Fragment) OS=Corethrella appendiculata PE=2 SV=1
A0A126GUR4_DROME	Eukarya	A	3.10E-24	96.2	0.3	Oskar, isoform D OS=Drosophila melanogaster GN=osk PE=4 SV=1
A0A059PGF2_9MUSC	Eukarya	A	3.10E-24	96.2	0.5	GA10627 (Fragment) OS=Drosophila pseudoobscura GN=GA10627 PE=4 SV=1
K4MTL4_GRYBI	Eukarya	A	1.20E-23	94.2	0.2	Oskar OS=Gryllus bimaculatus PE=2 SV=1
A0A0C9QHR7_9HYME	Eukarya	A	5.40E-22	89	0	Osk protein OS=Fopius arisanus GN=osk PE=4 SV=1
A0A059PF64_9MUSC	Eukarya	A	1.90E-19	80.8	0.7	GA10627 (Fragment) OS=Drosophila pseudoobscura GN=GA10627 PE=4 SV=1
A0A0J7KH44_LASNI	Eukarya	A	1.40E-13	61.9	0	Maternal effect protein oskar (Fragment) OS=Lasius niger GN=RF55_10783 PE=4 SV=1
E2BYH0_HARSA	Eukarya	A	4.00E-12	57.3	0	Putative uncharacterized protein OS=Harpegnathos saltator GN=EAI_08923 PE=4 SV=1
A0A0J6BBM7_BREBE	Bacteria	F	3.10E-10	51.2	0	Lysophospholipase OS=Brevibacillus brevis GN=AB432_04505 PE=4 SV=1
A0A0H0SJ00_9BACL	Bacteria	F	9.60E-10	49.6	0	Lysophospholipase OS=Brevibacillus formosus GN=AA984_15375 PE=4 SV=1
E9I8K8_SOLIN	Bacteria	A	5.90E-09	47.1	1	Putative uncharacterized protein (Fragment) OS=Solenopsis invicta GN=SINV_16199 PE=4 SV=1
J3A568_9BACL	Bacteria	F	5.80E-08	43.9	0	Lysophospholipase L1-like esterase OS=Brevibacillus sp. BC25 GN=PMI05_03395 PE=4 SV=1

G2HS43_9PROT	Bacteria	P	1.10E-07	43	1	Lipolytic protein OS=Arcobacter sp. L GN=ABLL_2651 PE=4 SV=1
E6L4E3_9PROT	Bacteria	P	1.70E-07	42.4	0.7	Lipolytic protein OS=Arcobacter butzleri JV22 GN=HMPREF9401_1319 PE=4 SV=1
R5GT16_9FIRM	Bacteria	F	2.60E-07	41.8	0	GDSL-like protein OS=Eubacterium sp. CAG:786 GN=BN782_00012 PE=4 SV=1
A0A078KJ49_9FIRM	Bacteria	F	3.40E-07	41.4	0	Uncharacterized protein OS=[Clostridium] cellulosi GN=CCDG5_0508 PE=4 SV=1
A8EWS4_ARCB4	Bacteria	P	3.80E-07	41.3	0.7	Lipolytic enzyme, GDSL domain OS=Arcobacter butzleri (strain RM4018) GN=Abu_2183 PE=4 SV=1
S5PEQ8_9PROT	Bacteria	P	4.00E-07	41.2	0.4	Lipolytic enzyme, GDSL domain protein OS=Arcobacter butzleri 7h1h GN=A7H1H_2115 PE=4 SV=1
A0A0G9K3L5_9PROT	Bacteria	P	4.00E-07	41.2	0.6	Lipolytic protein OS=Arcobacter butzleri L348 GN=AA20_04280 PE=4 SV=1
R6T7B3_9BACE	Bacteria	B	4.80E-07	41	0	GDSL-like protein OS=Bacteroides sp. CAG:770 GN=BN777_00744 PE=4 SV=1
A0A0A6S1U2_STRUB	Bacteria	F	4.90E-07	40.9	0.2	Acylneuraminate cytidylyltransferase OS=Streptococcus uberis GN=NC01_08240 PE=4 SV=1
W3AC50_9BACL	Bacteria	F	5.50E-07	40.8	0.3	Uncharacterized protein OS=Planomicrobium glaciei CHR43 GN=G159_16940 PE=4 SV=1
A0A0M1UPT0_9PROT	Bacteria	P	5.50E-07	40.8	0.4	Lipolytic protein OS=Arcobacter butzleri ED-1 GN=ABED_1978 PE=4 SV=1
U6EVC2_CLOTA	Bacteria	F	6.30E-07	40.6	0.7	Platelet activating factor acetylhydrolase-likeprotein OS=Clostridium tetani 12124569 GN=BN906_00617 PE=4 SV=1
A0A098EIL2_9BACL	Bacteria	F	7.90E-07	40.3	0.1	Multifunctional acyl-CoA thioesterase I and protease I and lysophospholipase L1 OS=Planomicrobium sp. ES2 GN=BN1080_01055 PE=4 SV=1
A0A069S7Q5_9PORP	Bacteria	B	1.10E-06	39.9	0	Uncharacterized protein OS=Parabacteroides distasonis str. 3776 Po2 i GN=M090_4091 PE=4 SV=1
Q897X6_CLOTE	Bacteria	F	1.20E-06	39.7	0.8	Platelet activating factor acetylhydrolase-like protein OS=Clostridium tetani (strain Massachusetts / E88) GN=CTC_00594 PE=4 SV=1
A0A0J9FZD4_9PORP	Bacteria	B	1.30E-06	39.6	0	Uncharacterized protein OS=Parabacteroides sp. D26 GN=HMPREF1000_00856 PE=4 SV=1
K9WJ28_9CYAN	Bacteria	C	1.60E-06	39.3	1.2	Lysophospholipase L1-like esterase OS=Microcoleus sp. PCC 7113 GN=Mic7113_4114 PE=4 SV=1
A0A0G1KN57_9BACT	Bacteria	?	3.70E-06	38.1	0	Secreted protein OS=candidate division WWE3 bacterium GW2011_GWC2_44_9 GN=UW82_C0006G0015 PE=4 SV=1
R5VMZ1_9FIRM	Bacteria	F	5.00E-06	37.7	0.1	GDSL-like protein OS=Firmicutes bacterium CAG:631 GN=BN742_01282 PE=4 SV=1
A0A0L0WAR6_CLOPU	Bacteria	F	7.20E-06	37.2	0.4	Lysophospholipase L1 OS=Clostridium purinilyticum GN=CLPU_6c00720 PE=4 SV=1
R5S0B3_9BACE	Bacteria	B	7.30E-06	37.2	0	GDSL-like protein OS=Bacteroides sp. CAG:545 GN=BN702_00435 PE=4 SV=1

A0A073IAZ3_9PORP	Bacteria	B	7.60E-06	37.1	0	Uncharacterized protein OS=Porphyromonas sp. 31_2 GN=HMPREF1002_01104 PE=4 SV=1
R4JC30_9BACT	Bacteria	?	7.90E-06	37.1	0.1	Uncharacterized protein OS=uncultured bacterium BAC25G1 GN=metaSSY_00600 PE=4 SV=1
A0A0C1UEU0_9CLOT	Bacteria	F	7.90E-06	37	0.8	GDSL-like Lipase/Acylhydrolase family protein OS=Clostridium argentinense CDC 2741 GN=U732_2423 PE=4 SV=1
R5LL12_9FIRM	Bacteria	F	1.00E-05	36.7	0	GDSL-like protein OS=Eubacterium sp. CAG:115 GN=BN470_02036 PE=4 SV=1
A0A0E3QN36_METBA	Archaea	Eu	1.20E-05	36.5	0.2	Putative tesA-like protease OS=Methanosarcina barkeri str. Wiesmoor GN=MSBRW_2234 PE=4 SV=1
B7KKA6_CYAP7	Bacteria	C	1.60E-05	36.1	0.3	Lipolytic protein G-D-S-L family OS=Cyanothece sp. (strain PCC 7424) GN=PCC7424_2577 PE=4 SV=1
R2P1Z4_9ENTE	Bacteria	F	1.60E-05	36	0.1	Uncharacterized protein OS=Enterococcus raffinosus ATCC 49464 GN=UAK_02837 PE=4 SV=1
A0A072Y8N5_9CLOT	Bacteria	F	1.80E-05	35.9	1.5	Acetylhydrolase OS=Clostridium sp. K25 GN=Z957_08245 PE=4 SV=1
K8GFE2_9CYAN	Bacteria	C	2.00E-05	35.8	0.1	Lysophospholipase L1-like esterase OS=Oscillatoriales cyanobacterium JSC-12 GN=OsccyDRAFT_3941 PE=4 SV=1
A0A095ZDI3_9FIRM	Bacteria	F	2.00E-05	35.8	0.3	Uncharacterized protein OS=Tissierellia bacterium S7-1-4 GN=HMPREF1634_08565 PE=4 SV=1
E1YW67_9PORP	Bacteria	B	2.20E-05	35.6	0	GDSL-like protein OS=Parabacteroides sp. 20_3 GN=HMPREF9008_00759 PE=4 SV=1
A0A0M0WNM0_9BACI	Bacteria	F	2.60E-05	35.4	0.4	Lipase OS=Bacillus sp. FJAT-21351 GN=AMS61_13120 PE=4 SV=1
A0A0G3CFD7_METBA	Archaea	Eu	2.80E-05	35.3	0.1	GDSL family lipase/acylhydrolase OS=Methanosarcina barkeri CM1 GN=MCM1_0752 PE=4 SV=1
F6DQC0_DESRL	Bacteria	F	3.40E-05	35	0	Lipolytic protein G-D-S-L family OS=Desulfotomaculum ruminis (strain ATCC 23193 / DSM 2154 / NCIB 8452 / DL) GN=Desru_1430 PE=4 SV=1
D5DGY9_BACMD	Bacteria	F	3.50E-05	35	0.5	Lipase/Acylhydrolase (GDSL) OS=Bacillus megaterium (strain DSM 319) GN=BMD_3140 PE=4 SV=1
A5N8N5_CLOK5	Bacteria	F	3.70E-05	34.9	1.6	Uncharacterized protein OS=Clostridium kluyveri (strain ATCC 8527 / DSM 555 / NCIMB 10680) GN=CKL_1624 PE=4 SV=1
R7ADB4_9BACE	Bacteria	F	3.90E-05	34.8	0.2	Uncharacterized protein OS=Bacteroides pectinophilus CAG:437 GN=BN656_00903 PE=4 SV=1
A0A0H1UPZ9_STRAG	Bacteria	F	4.30E-05	34.7	0.2	Acylneuraminate cytidylyltransferase OS=Streptococcus agalactiae GN=WA03_09270 PE=4 SV=1
A0A094AE00_9PEZI	Eukarya	As	0.00036	31.7	0.1	Uncharacterized protein (Fragment) OS=Pseudogymnoascus sp. VKM F-4281 (FW-2241) GN=V493_03380 PE=4 SV=1
A0A0L1HYX4_9PLEO	Eukarya	As	0.00066	30.9	0	Carbohydrate esterase family 3 protein OS=Stemphylium lycopersici GN=TW65_91054 PE=4 SV=1

G2QGB0_MYCTT	Eukarya	As	0.00074	30.7	0.1	Carbohydrate esterase family 3 protein OS=Myceliophthora thermophila (strain ATCC 42464 / BCRC 31852 / DSM 1799) GN=MYCTH_53698 PE=4 SV=1
E3RJZ5_PYRTT	Eukarya	As	0.0014	29.8	0	Putative uncharacterized protein OS=Pyrenophora teres f. teres (strain 0-1) GN=PTT_08513 PE=4 SV=1
G2QVW9_THITE	Eukarya	As	0.0066	27.7	0.1	Carbohydrate esterase family 3 protein OS=Thielavia terrestris (strain ATCC 38088 / NRRL 8126) GN=THITE_2042744 PE=4 SV=1
G0S9F4_CHATD	Eukarya	As	0.01	27.1	0	Putative uncharacterized protein OS=Chaetomium thermophilum (strain DSM 1495 / CBS 144.50 / IMI 039719) GN=CTHT_0045680 PE=4 SV=1

Supplementary File 1E: List of genomes analyzed for codon use.

This table lists the 17 genomes that were downloaded and analyzed for codon use as described in "*Selection of sequences for codon use analysis*" in Methods. All genomes can be downloaded from https://www.ncbi.nlm.nih.gov/genome/browse#!/overview/. The table lists the species name (*Species*), family (*Family*) and Order (*Order*), NCBI genome accession number (*Genome ID*), and the *oskar* NCBI Nucleotide accession number (*oskar Nucleotide ID*).

**Species**	**Family**	**Order**	**Genome ID**	**Oskar Nucleotide ID¬†**
Drosophila melanogaster	Drosophilidae	Diptera	GCA_001014345.1	NM_169248.4
Nasonia vitripennis	Pteromalidae	Hymenoptera	GCA_000002325.2	HM535628.1
Culex quinquefasciatus	Culicidae	Diptera	GCA_000209185.1	EU517695.1
Drosophila virilis	Drosophilidae	Diptera	GCA_000005245.1	L22556.1
Ceratitis capitata	Tephritidae	Diptera	GCA_000347755.4	LOC101450245
Musca domestica	Muscidae	Diptera	GCA_000371365.1	LOC101890691
Acromyrmex echinatior	Formicidae	Hymenoptera	GCA_000204515.1	LOC105147973
Harpegnathos saltator	Formicidae	Hymenoptera	GCA_000147195.1	LOC105187957
Bactrocera dorsalis	Tephritidae	Diptera	GCA_000789215.2	LOC105232054
Fopius arisanus	Braconidae	Hymenoptera	GCA_000806365.1	LOC105267990
Athalia rosae	Tenthrendinidae	Hymenoptera	GCA_000344095.2	LOC105692731
Orussus abietinus	Orussidae	Hymenoptera	GCA_000612105.2	LOC105696794
Stomoxys calcitrans	Muscidae	Diptera	GCA_001015335.1	LOC106086381
Bactrocera oleae	Tephritidae	Diptera	GCA_001188975.2	LOC106622417
Copidosoma floridanum	Encyrtidae	Hymenoptera	GCA_000648655.2	LOC106642594
Polistes canadensis	Vespidae	Hymenoptera	GCA_001313835.1	LOC106790143
Neodiprion lecontei	Diprionidae	Hymenoptera	GCF_001263575.1	LOC107223453
