# Supplementary material for: Bacterial contribution to genesis of the novel germ line determinant oskar
Source: eLife. 2020 Feb 24;9:e45539. doi: 10.7554/eLife.45539 (PMC7250577; doi:10.7554/eLife.45539)
Supplement: Transparent reporting form [file elife-45539-transrepform.docx]

***eLife’s* transparent reporting form**

We encourage authors to provide detailed information *within their submission* to facilitate the interpretation and replication of experiments. Authors can upload supporting documentation to indicate the use of appropriate reporting guidelines for health-related research (see [EQUATOR Network](http://www.equator-network.org/%20)), life science research (see the [BioSharing Information Resource](https://biosharing.org/)), or the [ARRIVE guidelines](http://www.plosbiology.org/article/info:doi/10.1371/journal.pbio.1000412) for reporting work involving animal research. Where applicable, authors should refer to any relevant reporting standards documents in this form.

If you have any questions, please consult our Journal Policies and/or contact us: [editorial@elifesciences.org](mailto:editorial@elifesciences.org).

**Sample-size estimation**

- You should state whether an appropriate sample size was computed when the study was being designed
- You should state the statistical method of sample size computation and any required assumptions
- If no explicit power analysis was used, you should describe how you decided what sample (replicate) size (number) to use

Please outline where this information can be found within the submission (e.g., sections or figure legends), or explain why this information doesn’t apply to your submission:

The relevant sample sizes for the analyses presented here are the numbers of *oskar* sequences aligned for use in phylogenetic and codon use analyses. The number of sequences we used was determined based on data availability in public databases at the time of the study.

All sequences found in the public databases at the time of the study that met our search criteria were used. Details of how we performed the search are described in the **Materials and Methods** sections *BLAST searches of oskar* and *Hidden Markov Model (HMM) generation and alignments of the OSK and LOTUS domain.*

For the codon use analyses, all publicly available insect genomes that had at least 8,000 gene annotations and possessed an *oskar* sequence according to our search criteria described above, were used. Details of how we selected these sequences are provided in the **Materials and Methods** section *Selection of sequences for codon use analysis.*

No power analysis or sample size computation was used. Instead, we used all data available at the time of the study. This study did not generate novel primary data, but rather used available sequence information.

**Replicates**

- You should report how often each experiment was performed
- You should include a definition of biological versus technical replication
- The data obtained should be provided and sufficient information should be provided to indicate the number of independent biological and/or technical replicates
- If you encountered any outliers, you should describe how these were handled
- Criteria for exclusion/inclusion of data should be clearly stated
- High-throughput sequence data should be uploaded before submission, with a private link for reviewers provided (these are available from both GEO and ArrayExpress)

Please outline where this information can be found within the submission (e.g., sections or figure legends), or explain why this information doesn’t apply to your submission:

No primary data generating experiments were performed, and as such the concept of experimental biological or technical replication does not apply here.

However, we used heuristic computational methods that required bootstrapping. For all such methods (RaxML tree and SOWHAT) we used 1000 bootstraps.

This information is found in the **Materials and Methods** sections *Phylogenetic* *Analysis*, and in the **Supplementary Information Methods** section *Statistical Analysis of Tree Topology*.

**Statistical reporting**

- Statistical analysis methods should be described and justified
- Raw data should be presented in figures whenever informative to do so (typically when N per group is less than 10)
- For each experiment, you should identify the statistical tests used, exact values of N, definitions of center, methods of multiple test correction, and dispersion and precision measures (e.g., mean, median, SD, SEM, confidence intervals; and, for the major substantive results, a measure of effect size (e.g., Pearson's r, Cohen's d)
- Report exact p-values wherever possible alongside the summary statistics and 95% confidence intervals. These should be reported for all key questions and not only when the p-value is less than 0.05.

Please outline where this information can be found within the submission (e.g., sections or figure legends), or explain why this information doesn’t apply to your submission:

For each statistical test performed, we report the p-value and the name of the test in the figure legend. Moreover, for transparency we provide the iPython notebook where each test was performed within the Supplementary Information Files. All tests were performed using the Python scipy stats module, and which notebook was used for each test is found in **Materials and Methods** sections *Fitting a linear model of codon use*, *Calculation of cosine distance* and *Calculation and analysis of the codon use Z score*.

For all tests, the number of sequences tested is reported in the corresponding **Materials and Methods** section of the manuscript.

(For large datasets, or papers with a very large number of statistical tests, you may upload a single table file with tests, Ns, etc., with reference to sections in the manuscript.)

**Group allocation**

- Indicate how samples were allocated into experimental groups (in the case of clinical studies, please specify allocation to treatment method); if randomization was used, please also state if restricted randomization was applied
- Indicate if masking was used during group allocation, data collection and/or data analysis

Please outline where this information can be found within the submission (e.g., sections or figure legends), or explain why this information doesn’t apply to your submission:

No group allocation was performed in this study.

**Additional data files (“source data”)**

- We encourage you to upload relevant additional data files, such as numerical data that are represented as a graph in a figure, or as a summary table
- Where provided, these should be in the most useful format, and they can be uploaded as “Source data” files linked to a main figure or table
- Include model definition files including the full list of parameters used
- Include code used for data analysis (e.g., R, MatLab)
- Avoid stating that data files are “available upon request”

Please indicate the figures or tables for which source data files have been provided:

For all figures, the raw data are provided either directly in table format, or as a file of the corresponding format (HMM, Trees, Alignments, BLAST search results).

All figures in the manuscript were generated using Python Seaborn, Matplotlib and ETE3. All scripts generating the figures for this manuscript, as well as standalone scripts, are provided as iPython notebooks hosted in the GitHub repository https://github.com/extavourlab/Oskar_HGT . The corresponding notebooks and scripts used are described in the **Materials and Methods** section of the manuscript.

Finally, the raw data used were downloaded from public databases as described in the **Materials and Methods** sections *BLAST searches of oskar* and *Hidden Markov Model (HMM) generation and alignments of the OSK and LOTUS domains*. Snapshots of the raw data files are available in the supplementary data. If not, they are downloaded by the provided scripts from those databases. All the unique corresponding identifiers of sequences used in this study are provided in Supplementary Tables S2, S3, and S4, which are all referred to in the main text and the Methods.
